# Understanding Period Poverty: Socio-Economic Inequalities in Menstrual Hygiene Management in Eight Low- and Middle-Income Countries

**DOI:** 10.3390/ijerph18052571

**Published:** 2021-03-04

**Authors:** Laura Rossouw, Hana Ross

**Affiliations:** 1 School of Economics and Finance, University of the Witwatersrand, Johannesburg 2193, South Africa; 2 School of Economics, University of Cape Town, Cape Town 7701, South Africa; hzarub1@yahoo.com

**Keywords:** menstrual health, menstrual hygiene management, inequality, water and sanitation, gender, environmental health, sanitary pads

## Abstract

Menstrual hygiene management and health is increasingly gaining policy importance in a bid to promote dignity, gender equality and reproductive health. Effective and adequate menstrual hygiene management requires women and girls to have access to their menstrual health materials and products of choice, but also extends into having private, clean and safe spaces for using these materials. The paper provides empirical evidence of the inequality in menstrual hygiene management in Kinshasa (DRC), Ethiopia, Ghana, Kenya, Rajasthan (India), Indonesia, Nigeria and Uganda using concentration indices and decomposition methods. There is consistent evidence of wealth-related inequality in the conditions of menstrual hygiene management spaces as well as access to sanitary pads across all countries. Wealth, education, the rural-urban divide and infrastructural limitations of the household are major contributors to these inequalities. While wealth is identified as one of the key drivers of unequal access to menstrual hygiene management, other socio-economic, environmental and household factors require urgent policy attention. This specifically includes the lack of safe MHM spaces which threaten the health and dignity of women and girls.

## 1. Introduction

Improving the menstrual health of women and girls is increasingly gaining policy importance in a bid to promote dignity, gender equality and reproductive health. Menstrual health management (MHM) consists of having access to clean absorbent materials, but also extends into having private and safe spaces for using these materials. Effective and adequate menstrual hygiene management requires women and girls to have access to menstrual health (MH) materials and products of sufficient quality and quantity to allow them to cleanly, safely, and comfortably manage and collect their menses [1].

Health education and identification and treatment of menstrual disorders will also enable women and girls to safely and appropriately manage their menstrual health [2]. Yet, the omnipresent stigmatization of menstruation and entrenched social norms in some parts of the world limit the adequate support to menstruating persons and results in MHM being a multi-sectoral policy challenge, affecting sexual and reproductive health, schooling and education, water, sanitation and hygiene (WASH), and more. Improving MHM is further complicated by the fact that access to MH products and safe, clean and private menstrual hygiene spaces are often not uniform across socio-economic status and geography.

The existing research links unhygienic conditions for using, cleaning and drying MH products to reproductive tract infections [3,4] and points to cases of the economically vulnerable having risky transactional sex for sanitary pads [5,6]. Qualitative studies describe how fear and shame around menstrual hygiene as a result of stigmatization inhibit mobility and participation in society, which results in social isolation [7,8]. Even though the early studies of the impact of poor MHM on labour market and educational outcomes produced mixed results [7,9,10,11], more recent evidence points to the quantitative association between poor MHM and school non-attendance [8,12]. The inconsistent findings with respect to human capital formation do not necessarily mean that MHM does not have economic impact, because the topic has not been properly studied [13] and it is challenging to measure and quantify the impact of MHM on economic performance.

Given the potential health, social and economic consequences of ineffective MHM, unequal access to it will perpetuate existing socio-economic inequalities within society. For this reason, we aim to measure the magnitude of inequality in MHM by socio-economic status in eight low- and middle-income countries (Democratic Republic of Congo (Kinshasa), Ethiopia, Ghana, Kenya, India (Rajahstan), Indonesia, Nigeria and Uganda). We also investigate factors contributing to these inequalities to help to identify policy interventions that could address them.

The manner in which the market for MH products has developed has shaped the demand for sanitary pads as aspirational in managing menstrual hygiene [14], even though the use of these products poses affordability and environmental concerns [15,16]. The penetration of other products like tampons or environmentally friendly reusable menstrual cups is still low in LMICs. There is evidence that training and peer support significantly increases uptake and acceptability of menstrual cups [17]. Cloth usage is often framed as an unhygienic option. As Mahajan (2019) points out, in truth absorbent and clean cloth in itself is a traditional and affordable MH product. However, a lack of access to adequate WASH facilities and the potential shame of washing used cloth in public or in front of family members often result in poor maintenance practices and potentially concerns around hygiene [14].

In order to reflect these de facto practices in the current demand for MH products, we define access to menstrual hygiene products as access to sanitary pads, due to the aspirational nature of these products. We measure this access by self-reported use, even though we are aware that utilization may not necessarily reflect the preferred choice of MH product.

In addition to the choice of MH product, we also explore the inequality in access to clean, private, safe, and lockable MHM spaces with water and soap. We look beyond wealth as a determinant of access and point to household and environmental attributes which needs to be addressed for adequate MHM. Drawing on the Performance Monitoring and Accountability 2020 (PMA2020) data, we use concentration indices and decomposition methods to measure the extent of and contributors to MHM inequality in the countries studied.

## 2. Materials and Methods

### 2.1. Data

There are very few nationally representative datasets that collect information on menstrual health practices. In that sense, the Performance Monitoring and Accountability 2020 (PMA2020) survey, which employs a multi-stage cluster sampling design and utilizes enumeration areas drawn from a master sampling frame provided by statistical agencies, is unique [18]. PMA2020 collected data in selected LMICs in Africa and Asia, namely Burkina Faso, Ivory Coast, Democratic Republic of Congo (Kinshasa), Ethiopia, Ghana, Kenya, India (Rajasthan), Indonesia, Nigeria, Niger and Uganda. We excluded Burkina Faso, Ivory Coast and Niger from our analysis due to the comparability of MHM data and varying wealth measures. The lack of income variation in Niger and Burkina Faso resulted in wealth measured in tertiles rather than quintiles, so it was excluded. MHM indicators were not collected for Ivory Coast.

We focused on data collected among women aged 15 to 49 who experienced a menstrual period in the last three months and reported on their MHM as well as other behaviors related to family planning, reproduction, fertility and sexual activity. This section of the survey was administered by a female enumerator in auditory privacy to protect the participants and to secure an adequate response rate [2]. Our analytical sample was restricted to those with no missing observations. Sample statistics are provided in Table 1. The difference between the MHM sample and the analytical sample is the result of missing observations in the dependent (MHM measures and wealth) and independent variables (place of residence, education, marital status, age, use of family planning, access to a flush toilet, and place to wash hands).

### 2.2. Menstrual Hygiene Management Data and Measures

During the survey, respondents were asked to list all MH products that they used during their last menstrual period. Respondents were able to list more than one type of MH product, so the categories are not mutually exclusive. Materials listed by women included sanitary pads, tampons, cloth, cotton wool, materials from nature, toilet paper, foam (e.g., from a mattress), a bucket, diapers, or nothing at all. Sanitary pads were a consistently large category across countries and are the focus of the analysis on access to products.

The MH product variable, *‘no sanitary pads’*, is a binary variable set equal to one if the respondent did not access sanitary pads and zero if they did. Therefore, the sanitary pad variable is shaped as a lack of access.

Respondents reported on access to safe, clean, and private spaces for changing MHM materials by answering the following question: “While managing your menstrual hygiene, was this place: clean, private, safe, able to be locked, supplied with water, supplied with soap”. Respondents chose from the list of these 6 options and we used their answers to create six binary variables coded as one if the respondent did not have access, zero otherwise. We also created a binary variable equal to one if the respondent did not have access to any of the six conditions, zero otherwise.

As a result, we have eight MHM measures for the analysis, namely lack of access to clean (1), private (2), safe (3), and lockable (4) MHM spaces, lack of water (5) and soap (6) at MHM spaces, access to none of these conditions (7), and did not have access to sanitary pads as a MHM product (8).

### 2.3. Wealth Measure

A measure of wealth is required to establish the extent of inequality in access to MHM driven by economic circumstances. Household level of wealth was derived from the ownership of various assets, building material used for the primary residence, and access to water and sanitation facilities. Wealth quintiles are then calculated and reported by the primary data collectors [27].

### 2.4. Concentration Index

The concentration index is a tool which can be used to quantify the extent of inequality in one outcome over the distribution of another outcome [28]. In our analysis, we use it to measure the extent of inequality in our eight MHM measures over the distribution of wealth.

The calculated index takes a value between minus and plus one. A negative (positive) value indicates that the MHM measure is less accessible to the poor (the non-poor). A value equal to zero indicates equality [29]. The higher the concentration index, the higher the level of inequality.

The standard concentration index can be written in various ways, and we adopt the formula as reported Wagstaff et al. [30] and Kakwani et al. [31] expressed as follows:

(1) $$C_{s}=\;\frac{2}{n\mu }\;\sum_{i=1}^{n}MHM_{mi}r_{i}-1$$

where $MHM_{mi}$ is the MHM variable m, µ is its mean, $r_{i}$ is the fractional rank of individual *i* in the wealth distribution from the relatively poorest to the relatively richest of population *n*.

Variations of concentration indices have been developed and used to accommodate the properties of the outcome measures [32]. Our measures of MHM are binary and bounded in nature, so the standard concentration index is problematic in measuring inequalities and requires some form of normalization [33]. As a result, we adopt the Erreygers’ corrected concentration index (CCI). The index, as proposed by Erreygers [34], is simplified for computation in their commentary on the index by Wagstaff [35] Wagstaff expresses the Erreygers’ CCI as follows:

(2) $$CCI_{E}=4\;\frac{\mu }{b-a}*C_{s}$$

where µ is the mean of the MHM variable, *b* is its upper limit, *a* its minimum, and $C_{s}$ the standard concentration index prior to Erreygers’ correction as illustrated in Equation (1).

### 2.5. Decomposition Analysis

We decompose the concentration index in order to determine to what extent the inequality in MHM measures can be explained by wealth itself, and how much can be explained by other socio-economic, household, and environmental factors. Following the methodology of Wagstaff, Van Doorslaer and Watanabe [30], we start with the linear relationship between the MHM measure *m* and its explanatory variables $x_{ik}$, which can be expressed as follows:

(3) $$MHM_{mi}=\;\beta_{0}+\;\sum_{k=1}^{K}\beta_{k}x_{ik}+\;\varepsilon_{mi}$$

where MHM is the MHM measure *m* for individual *i*, the Betas are the coefficients, $\varepsilon_{mi}$ is the error term and $x_{ik}$ is the set of *k* socio-economic, household and environmental factors for individual *i*. Drawing from Wagstaff, Van Doorslaer and Watanabe, the linear model given the relationship between $MHM_{mi}$ and $x_{ik}$ in Equation (3), the standard concentration index for $MHM_{m}$, can written as:

(4) $$C_{s}=\;\sum_{k=1}^{K}(\beta_{k}{\bar{x}}_{k}/\mu )C_{k}+\frac{2}{n}\;\sum_{i=1}^{n}\varepsilon_{mi}R_{i}{}_{k}/\mu \;$$

where ${\bar{x}}_{k}\;\mathrm{is}\;$ the mean of $x_{k}$, μ is the mean of $MHM_{m}$, $C_{k}$ is the concentration index for $x_{k}.$ $C_{k}$ will therefore tell us the level of inequality in variable $x_{k}$, which we want to understand in order to inform the extent to which this inequality affects inequality in MHM. The latter term is the generalized concentration index of the error term, and can be rewritten as $GC_{\varepsilon }/\mu$.

Taking into account that Equation (4) is for the standard concentration index, the equation is converted to suit an Erreygers’ CCI in applied research [36,37] as follows:

(5) $$CCI_{E}\left(MHM_{m}\right)=4(\sum_{k=1}^{K}\beta_{k}{\bar{x}}_{k}C\left(x_{k}\right)+GC_{\varepsilon })$$

This decomposition is made of several components. $\beta_{k}{\bar{x}}_{k}$ is the elasticity of factor $x_{k}$ to MHM measure changes, $C_{k}$ is the concentration index for $x_{k}\;$ and $GC_{\varepsilon }$ the unexplained error term. Using these components and Equation (3), we report on three sets of outcome measures with interpretive value.

- Beta: Firstly, we report on the Beta from Equation (3). The beta indicates the direction of the relationship between variable $x_{k}$ and the MHM measure. It therefore does not illustrate inequality, but the point change in the probability of the MHM measure associated with a unit change of each variable $x_{k}$.
- The Contribution rows show the absolute contribution of each explanatory variable to the overall wealth-related inequality in MHM measure m. This contribution is calculated as the product of the explanatory variable’s elasticity and own concentration index to the wealth index. The Contribution is greater if the explanatory variable’s own concentration index is larger, it has a higher mean or a greater Beta.
- The Contribution % row translates the absolute contribution into a percentage contribution.

This full set of results are available in the supplementary results and interpreted in the text. Figures representing Contribution % are included in the text for ease of interpretation. While Wagstaff and co-authors present one, oft-used method of decomposition, there has been several developments in the measurement and decomposition of health inequalities. A recent approach developed by Heckley, Gerdtham and Kessels uses a recentered influence function regression to decompose the inequalities into underlying explanatory variables [38]. Kessels and Erreygers introduced a structural equation modelling framework for the decomposition of rank-dependent indicators of socio-economic-related health inequalities [39]. More recently, Kessels and Erreygers [40] also developed an easily interpretable, direct regression-based decomposition approach. The fundamental aspect of the approach is that it focuses on the individual components of the health indicators rather than its influences, as other approaches have done. While the Wagstaff et al. method has often been described as one-dimensional [38,40], we use the dominant strategy to increase comparability with other studies which measure health inequalities using this methodology.

Estimating this equation does not produce analytical standard errors, so a bootstrapping technique taking into account the data’s sampling structure was used to generate standard errors for the absolute contribution of a factor to MHM inequality. Bootstrapping at 500 replications was applied. The method is described in the statistical literature [41,42], and widely applied in health inequality studies [37,43]. Similar to other studies [36,37], we use a generalized linear model (GLM) in the decomposition estimation. When the outcome variable (our various MHM measures) is binary, the GLM is considered less sensitive to the choice of reference group [44].

Concentration indices and decomposition approaches are used in the analysis to gauge the full distribution of menstrual hygiene management in a country, rather than focusing on the lower end of the distribution only as would be the case with regression-analysis. The approach allows us to understand why inequalities exist, rather than purely gauging what factors contribute to access to lack thereof. The concentration index allows us to measure the degree of wealth-related inequality. Furthermore, the regression-based decomposition analysis allows us to jointly assess how the wealth-related inequality in our explanatory variables, as well as its correlatory relationship to the MHM measures and their own mean contributes to MHM inequality. Therefore, the complex nature of the analysis allows us to quantify the relative contribution of each of the various inequalities in explaining wealth-related inequalities in MHM.

### 2.6. Independent Variables

The selection of independent variables (*x*) included in the decomposition analysis is based on the literature on the socio-economic barriers to safe and adequate MHM: the respondent’s place of residence (urban/rural), education, marital status, age, use of family planning, access to a flush toilet, and access to a place to wash their hands (this variable is missing for Nigeria). The use of family planning is a proxy for having access to women’s health services. It is also included to discern whether there is potential to use family planning centers to improve access to MHM products. Even though the access to a flush toilet (i.e., flush systems connected to sewerage, septic, or pit latrines) is used as a proxy for the access to sanitary facilities, this also reflects country-specific norms and infrastructure regarding sewerage systems and not necessarily the respondent’s household environment. The urban/rural variable is not included in the Kinshasa sample, given that it is purely urban.

The ranking variable (wealth quintiles) is also included in the decomposition analysis, in order to establish the contribution of wealth in itself to the inequality relative to the other factors. The practice of including the rank variable itself is also done by Wagstaff and co-authors in their seminal paper on health inequality decomposition [30], by O’Donnell and co-authors in their handbook on analyzing health equity [45] and is often performed in applied literature [36,37,43,46,47].

The decomposition analysis is performed for each country separately with female specific sample weights was done using Stata 16, and results are considered significant at a 5% level. The reason for a separate analysis for each country instead of performing a pooled analysis with country fixed effects is due to the construction of the wealth index. The wealth index is divided into wealth quintiles, which is based on the distribution of wealth within that country. Since wealth quintiles reflect the relative situation in each country, they are not comparable across countries. For instance, the top wealth quintile in Kinshasa may look completely different from the wealth quintile in Ethiopia due to differences in the wealth distribution.

## 3. Results

The summary statistics of our samples are presented in Table 2. Data can be interpreted in terms of percentages, as they are the mean of binary variables. We report on the results from the MHM measures, but the full set of results is available in the Supplementary Table S1. In all countries other than Ethiopia (51%), less than 20% of respondents identified their MHM spaces as not being clean. Women in Kinshasa (DRC) and Ethiopia reported a high degree of the lack of privacy (Kinshasa = 57%, Ethiopia = 31%) and safety (Kinshasa = 35%, Ethiopia = 51%) with regard to MHM spaces. An alarming share of women are not able to lock their MHM space: 75% in Kinshasa, 73% in Ethiopia, 50% in Rajasthan, 47% in Uganda, 35% in Ghana and Nigeria, 31% in Kenya, and 18% in Indonesia. In all countries other than Indonesia, MHM spaces lack soap and water, the most notably in Kinshasa where 83% and 84% of the sample does not have access to water or soap in their MHM spaces, respectively. With the exception of Indonesia (30%), more than 70% of our respondents do not have access to all six conditions for a safe, clean and private MHM space.

Access to sanitary pads varies significantly across countries. In Kinshasa, Kenya, Ghana, and Indonesia, only 17%, 14%, 10%, and 9% of women and girls report not using sanitary pads as a menstrual hygiene product, respectively. The non-use is higher in Rajasthan (54%), Ethiopia (41%), Nigeria (37%) and Uganda (36%).

We consistently find unequal access to MHM by wealth status. This is evident from the negative concentration indices across MHM factors and across countries (Table 3). Women and girls from households with less wealth are less likely to have access to clean, private, safe and lockable spaces for menstrual hygiene management, and are less likely to have access to soap and water than females living in wealthier households. These disparities are particularly pronounced in Ethiopia, Rajasthan and Nigeria. In Ethiopia, the concentration index for not having clean (CCI = −0.33), safe (CCI = −0.35) and lockable (CCI = −0.31) MHM spaces is large and statistically significant. In Rajasthan, all the factors other than the privacy condition has CCIs larger than −0.2. The most notable is that the poor in Rajasthan have significantly less access to lockable MHM spaces (CCI = −0.53). Similarly, Nigeria’s CCI for lockable and safe MHM spaces are −0.54 and −0.33, respectively. The concentrations indices for clean (CCI = −0.27) and private (CCI = −0.28) MHM spaces, with access to water (CCI = −0.29) and soap (CCI = −0.25) in Nigeria also indicate that less affluent women and girls are much more likely to lack these MHM sanitation conditions compared to their affluent counterparts.

The same trend is present when we assess access to sanitary pads. In Ethiopia (CCI = −0.63), Rajasthan (CCI = −0.45) and Nigeria (CCI = −0.59), the large and negative concentration indices for “No pads used” indicates that lack of access is concentrated among the poor. The inequalities are also present in the remaining countries of analysis, but to a lesser extent.

**Figure 1:**
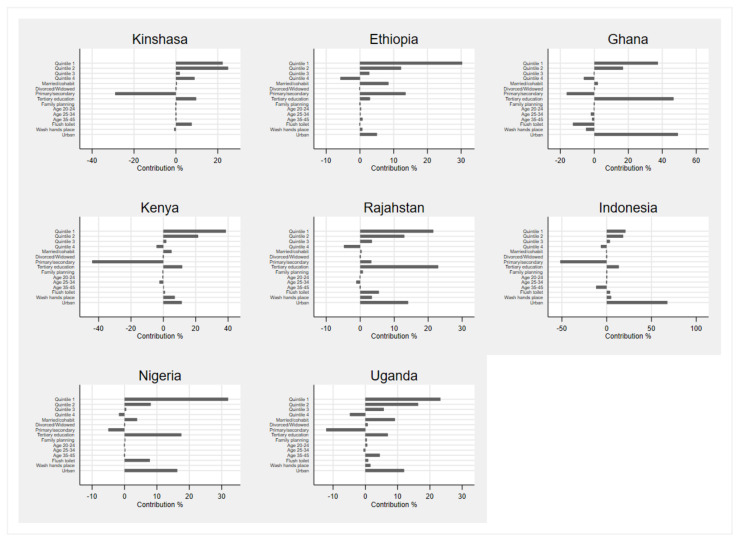
Contribution % of various factors to the CCIs of sanitary pad access.

**Figure 2:**
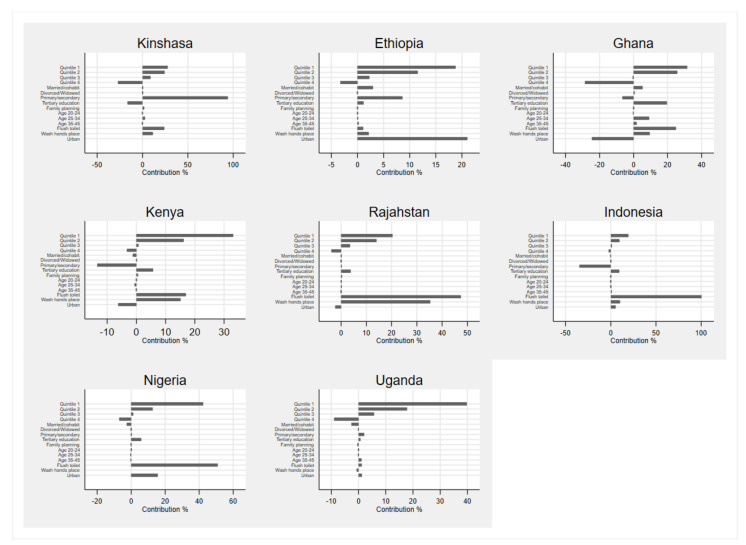
Contribution % of various factors to the CCIs of MHM conditions.

The decompositions of the CCIs are presented in Supplementary Tables S2 and S3, and discussed in the text. The contribution percentages of these tables are presented graphically in Figure 1 and Figure 2 for ease of interpretation.

The Betas in Supplementary Table S2 show that in most countries, women and girls with limited access to wealth and education, living in rural areas, and women older than 35 are consistently less likely to have access to sanitary pads. Women who are married and divorced are also less likely to access sanitary pads, but these results are only statistically significant in Ethiopia, Kenya and Nigeria.

Unsurprisingly, wealth itself is the biggest contributor to the overall wealth-related inequality in accessing sanitary pads in most countries (Figure 1 and Contribution % in Supplementary Table S2). The contribution of being in wealth quintile 1 (i.e., having the least wealth) contributed significantly to inadequate access for the poor: 22%, 30%, 37%, 39%, 22%, 21%, 32% and 23% contribution to the overall inequality in access to sanitary pads in Kinshasa, Ethiopia, Ghana, Kenya, Rajasthan, Indonesia, Nigeria and Uganda, respectively. Only in Ghana and Indonesia do we observe that the level of education (having tertiary education as opposed to no education) makes a bigger contribution. These account for 47% and 23% of the CCI in Ghana and Indonesia, respectively. The Betas on the urban-rural variable indicate that women living in urban areas are significantly more likely to have access to sanitary pads than their rural counterparts. Residing in rural areas increases the wealth-related sanitary pad inequality, especially in Indonesia where it is the biggest contributor at 68%.

When it comes to having access to the necessary MHM sanitation conditions (Figure 2 and Supplementary Table S3), we find more heterogeneity across countries. While wealth is an important contributor to the widening gap between the poor and the relatively wealthy, other factors also play important roles. In Kinshasa and Ethiopia, the education gap between those having no-schooling and those with Primary and Secondary School education is a statistically significant contributor to inequality, at 94% and 9%, respectively. In both these countries, by increasing the level of education of women and girls and removing inequalities in education, we could potentially narrow the gap in MHM inequalities. In all countries other than Ethiopia and Uganda, not having access to a flush toilet in their house or surrounds significantly contributes to the inequality in safe and adequate MHM conditions. The same is true for not having access to spaces where a person is able to wash her hands.

While the urban-rural divide is a crucial contributor to accessing sanitary pads across countries, the role of urban-rural residence is less clear when it comes to accessing safe and adequate MHM conditions. Living in an urban setting contributes significantly to inequality by 21%, 25%, 6%, 2%, 5%, 16% and 1% in Ethiopia, Ghana, Kenya, Rajasthan, Indonesia, Nigeria and Uganda, respectively. However, the effect is a mix of pro-poor and pro-affluent depending on the country. In Ghana, Kenya and Rajasthan, the urban-rural divide decreases the wealth gap in access to safe MHM space, while it does the opposite in Ethiopia, Indonesia, Nigeria, and Uganda.

## 4. Discussion

In this paper, we measure the level of unequal access to MHM in Kinshasa (DRC), Ethiopia, Ghana, Kenya, Rajasthan (India), Indonesia, Nigeria and Uganda, as well as their contributing factors. Ineffective MHM holds potential health, social and economic consequences. However, access to MH products and safe, clean and private menstrual hygiene spaces are often unequally distributed by socio-economic status, which in turn will perpetuate existing socio-economic inequalities within society.

There is consistent evidence of wealth-related inequality in the conditions of MHM spaces across all countries. These disparities are particularly pronounced in Ethiopia, Rajasthan and Nigeria. The most notable and worrisome inequality is in having access to lockable and safe MHM spaces. Across countries, women and girls from less wealthy households are less likely to access safe and lockable MHM spaces compared to those from wealthy households. Ensuring that all females have access to MHM spaces where they feel safe and empowered is non-negotiable. This is particularly worrisome given the prevalence and severe consequences of gender-based violence.

The planning and design of sanitation systems rarely incorporates or considers the needs and practices of menstruating women and girls [16]. Taboos and misconceptions around menstruation in the past often resulted in dealing with MHM in an unnoticeable manner, using restrictions or exclusions rather than creating the clean, safe and private spaces [13]. Sommer and co-authors (2013) identify the need for private MHM spaces as key, especially in environments where menstruation is still considered culturally taboo and menstruation dangerous. Privacy includes the ability to manage menstrual hygiene anonymously [16].

The unequal access to private, safe and clean MHM spaces is largely driven by differences in wealth, education, and the infrastructural limitations of the household. In all countries other than Ethiopia and Uganda, not having access to a flush toilet and a place to wash your hands, significantly contributes to the inequality of safe and adequate MHM conditions. We observe very different sanitary facilities across countries. In Kinshasa, Rajasthan and Indonesia, more than 50% of respondents report having access to some form of flush toilet. In Ethiopia and Uganda, this proportion is only 10% and 6%, respectively. Similar disparities exist for spaces to wash hands. These findings may be more representative of the country specific infrastructure and environment than the individuals’ socio-economic circumstances. For instance, the most common toilet facility in our data for Ethiopia and Uganda are pit latrines with no slab or an open pit latrine (60% of the women sampled in both countries). Such pit latrines without hand washing facilities are considered unhygienic [48]. Similar levels of poor infrastructure have been measured in other studies on Ethiopia [48] and Uganda [49], although the findings in Uganda in our study are lower compared to some other studies.

There are also clear wealth-related inequalities in access to MH products and materials. Across countries, those living in wealthier households are more likely to access sanitary pads than those living in less wealthy households. The use of insertable products is low across the countries in our sample and across LMICs in general. It is crucial to acknowledge that while the research question frames sanitary pads as the MH product of choice, this may not always be the case, because the choice of MH products is also influenced by cultural context. The use of absorbent and clean cloth as a MH product may not in itself pose a threat to health. However, poor maintenance of the cloth’s cleanliness due to limited WASH access may inhibit this maintenance [14].

Unequal wealth-related access to sanitary pads is driven by socio-economic indicators including less education and residing in a rural as opposed to an urban environment. These factors are likely to affect employment opportunities and incomes. In return, they determine the access to sanitary pads in the countries studied.

One policy intervention to improve access to MH products and remove wealth-related inequalities is the removal of taxes on menstrual hygiene products. Taxes on menstrual products constitute an implicit form of gender bias, given that they pose a financial constraint on women and not men, possibly perpetuating existing economic inequalities created by gendered wage gaps. Removing taxes on MH products is justified in the broader framework of removing gender bias in the taxation system [50]. Taxes on menstrual products create inequities along gender and socio-economic lines: while they are currently only borne by women, they also constitute a larger portion of the less affluent households’ budgets. The fiscal policy of removing MH product taxes can be a potentially effective tool to increase affordability of these products and reduce the economic burden on women and their families.

However, the effectiveness of tax removal in reducing the retail price of MH products is not clear [51]. Evidence suggests that a lack of market competition and the complicated price structure of MH products may impede the effectiveness of this policy [52]. The impact on affordability aside, the advocacy campaigns addressing menstrual hygiene taxation have been key in initiating the conversation around the MHM and addressing the stigmatisation around menstruation [53]. A future research priority for effective MHM should include data collection and evaluation of MH policies in order to assess their effectiveness should they be applied broadly.

A major limitation of the study is that we are unable to distinguish between the quality and frequency of products being used. We risk underestimating the socio-economic divisions by not being able to differentiate between those who have an inadequate or adequate supply of MH products. A recent study using the PMA2020 data found that 26.4% of those exclusively using sanitary pads report unmet material needs. This statistic was skewed towards disadvantaged groups [2]. This may be the reason for women and girls alternating between products, depending on whether they are at home or in public, and for multiple options listed by the participants of the PMA2020 survey.

Another concern is the presence of underreporting and missing observations in household surveys. These methodological issues would be particularly pronounced in countries where there is still much stigma around MHM. For instance, an analysis focusing on South Africa found that approximately 50% of the relevant participants Living Conditions Survey had underreported MH products used [54].

Going forward, it is crucial to remember that MHM spaces and MH products or materials are one component of a larger challenge to improve MHM globally. Van Eijk et al. (2016) identify three broad areas for adequate MHM, including individual knowledge, the material environment and the social environment. Individual knowledge includes knowledge and normalization of the biological process of menstruation, and the social environment refers to addressing the broader taboos, myths, and stigmatization around MHM. The material environment includes both the materials to collect or absorb menstrual blood as well as the facilities to privately and hygienically manage menstruation. While this paper addresses the material environment, it is crucial to address and facilitate all three areas [15]. These areas are also interrelated. As Sommer et al. (2013) writes, individual knowledge around MHM is necessary for women and girls to use and take up water and sanitation systems via their empowerment and confidence around MHM [16]. While knowledge and self-confidence on how to navigate the structural realities of their MHM environment is key, the social environment around MHM may in turn influence these structural realities.

Key issues on the topic of MHM were mentioned throughout the manuscript and contributes to the agenda of urgent research on MHM. One of these is to reshape the framing of aspirational MH products in research questions of intervention development. The choice of MH product should be culturally appropriate, suit the needs and preferences of users and allow for private and clean use within their available MHM spaces.

Perhaps the most pressing and omnipresent issue concerning inefficient MHM is its ongoing stigmatization. The stigmatization disallows an open discussion of problems related to women and girls’ experiences of MHM, and without the dialogue, it is difficult to identify solutions. While great strides have been made to address stigma, it remains an urgent research priority.

## 5. Conclusions

The results show that while wealth is one of the key drivers of unequal access to MHM, other socio-economic, environmental and household factors require urgent policy attention. The lack of safe MHM spaces and unequal access to sanitary pads for poor women and girls in the LMICs affects their health and dignity and needs to be prioritized.

Despite recent attention, there is still a dearth of rigorous evaluations and research investigating the effectiveness of policy interventions to improve menstrual health. This lack of evidence highlights the need for more research funding focusing on MHM interventions [1,55].

## Figures and Tables

**Table 1:** PMA2020 sample statistics.

	MHM Sample	Analytical Sample	Date Collected
Kinshasa (DRC) [19]	2125	2102	2017
Ethiopia [20]	4954	4814	2017
Ghana [21]	2935	2861	2016
Kenya [22]	4573	4478	2017
Rajasthan (India) [23]	5133	5018	2017
Indonesia [24]	8274	8122	2016
Nigeria [25]	8469	8121	2018
Uganda [26]	2798	2736	2017

**Table 2:** Summary statistics.

		DRC (Kinshasa)	Ethiopia	Ghana	Kenya	India (Rajasthan)	Indonesia	Nigeria	Uganda
		Mean	Mean	Mean	Mean	Mean	Mean	Mean	Mean
Condition of main place for managing menstrual hygiene	Not clean	0.158	0.511	0.137	0.125	0.187	0.054	0.134	0.091
	No private	0.565	0.306	0.152	0.128	0.112	0.073	0.171	0.142
	Not safe	0.348	0.512	0.183	0.149	0.202	0.089	0.187	0.218
	Cannot lock	0.745	0.727	0.352	0.314	0.493	0.183	0.348	0.465
	No water	0.826	0.549	0.583	0.608	0.353	0.083	0.59	0.418
	No soap	0.838	0.631	0.561	0.674	0.39	0.135	0.618	0.425
	Does not have all six conditions	0.961	0.883	0.733	0.752	0.629	0.294	0.738	0.701
Materials used	No pads used	0.168	0.41	0.103	0.136	0.535	0.087	0.367	0.357
N		2102	4814	2861	4478	5018	8122	8121	2736

**Table 3:** Wealth-related inequalities in MHM: Evidence from CCIs.

		Kinshasa	Ethiopia	Ghana	Kenya	Rajasthan	Indonesia	Nigeria	Uganda
		CCI	CCI	CCI	CCI	CCI	CCI	CCI	CCI
Condition of main place for managing menstrual hygiene	Not clean	−0.06 ***	−0.334 ***	−0.075 ***	−0.138 ***	−0.238 ***	−0.091 ***	−0.268 ***	−0.117 ***
	No private	−0.028	−0.176 ***	−0.156 ***	−0.095 ***	−0.1 ***	−0.138 ***	−0.283 ***	−0.105 ***
	Not safe	−0.147 ***	−0.35 ***	−0.133 ***	−0.135 ***	−0.251 ***	−0.13 ***	−0.317 ***	−0.158 ***
	Cannot lock	−0.221 ***	−0.307 ***	−0.191 ***	−0.405 ***	−0.529 ***	−0.276 ***	−0.544 ***	−0.467 ***
	No water	−0.05 ***	−0.23 ***	−0.092 ***	−0.25 ***	−0.338 ***	−0.104 ***	−0.285 ***	−0.293 ***
	No soap	−0.032 *	−0.168 ***	0.021	−0.184 ***	−0.308 ***	−0.124 ***	−0.249 ***	−0.246 ***
	Does not have all six conditions	−0.037 ***	−0.16 ***	−0.127 ***	−0.235 ***	−0.513 ***	−0.312 ***	−0.385 ***	−0.403 ***
Materials used	No pads used	−0.15 ***	−0.627 ***	−0.18 ***	−0.198 ***	−0.447 ***	−0.093 ***	−0.586 ***	−0.303 ***

*** *p* < 0.01, * *p* < 0.1.

## Data Availability

The secondary data was sourced by application from the Performance Monitoring and Accountability 2020 website, and is available from them upon request.

## Supplementary Materials

The following are available online at https://www.mdpi.com/1660-4601/18/5/2571/s1, Table S1: Full set of Summary Statistics, Table S2: Decomposition of the CCI of sanitary pad access, Table S3: Decomposition of the MHM conditions CCI.
