# Supplementary material for: Understanding Period Poverty: Socio-Economic Inequalities in Menstrual Hygiene Management in Eight Low- and Middle-Income Countries
Source: Int J Environ Res Public Health. 2021 Mar 4;18(5):2571. doi: 10.3390/ijerph18052571 (PMC7967348; doi:10.3390/ijerph18052571)
Supplement: Supplementary file 1 [file ijerph-18-02571-s001.pdf]

Supplementary Material

Table S1. Full set of Summary Statistics.

		DRC (Kinshasa)	Ethiopia	Ghana	Kenya	India (Rajasthan)	Indonesia	Nigeria	Uganda
		Mean	Mean	Mean	Mean	Mean	Mean	Mean	Mean
Marital Status	Never married	0.561	0.415	0.377	0.387	0.235	0.29	0.372	0.315
	Married/cohabitating	0.376	0.466	0.513	0.538	0.74	0.666	0.576	0.537
	Divorced/Widowed	0.063	0.119	0.11	0.075	0.025	0.044	0.052	0.148
	Total	=1	=1	=1	=1	=1	=1	=1	=1
Education	No schooling	0.019	0.247	0.146	0.041	0.364	0.013	0.171	0.09
	Primary or Secondary schooling	0.824	0.623	0.566	0.813	0.423	0.823	0.635	0.824
	Tertiary education	0.158	0.13	0.288	0.146	0.213	0.164	0.194	0.086
	Total	=1	=1	=1	=1	=1	=1	=1	=1
Currently using family planning		0.435	0.277	0.281	0.469	0.423	0.391	0.252	0.366
Age	Age 15-19	0.249	0.298	0.211	0.246	0.214	0.173	0.237	0.25
	Age 20-24	0.206	0.189	0.192	0.18	0.186	0.136	0.157	0.202
	Age 25-34	0.304	0.291	0.312	0.314	0.324	0.266	0.305	0.289
	Age 35-45	0.241	0.221	0.285	0.26	0.275	0.425	0.301	0.26
	Total	=1	=1	=1	=1	=1	=1	=1	=1
Access to a flush toilet of sorts		0.551	0.102	0.273	0.194	0.515	0.889	0.471	0.063
Self-report having access to place to wash hands		0.495	0.123	0.157	0.41	0.66	0.533	-	0.258
Residence	Urban	-	0.596	0.58	0.361	0.261	0.576	0.532	0.304
	Rural	-	0.404	0.42	0.639	0.739	0.424	0.468	0.696
	Total		=1	=1	=1	=1	=1	=1	=1
N		2102	4814	2861	4478	5018	8122	8121	2736

Table S2. Decomposition of the CCI of sanitary pad access.

		Kinshasa	Ethiopia	Ghana	Kenya	Rajasthan	Indonesia	Nigeria	Uganda
Quintile 1 (vs Quintile 5)	Beta	0.12***	0.44***	0.14***	0.19***	0.28***	0.068***	0.37***	0.18***
	Contribution	-0.03***	-0.19***	-0.07***	-0.08***	-0.10***	-0.02***	-0.19***	-0.07***
	Contribution %	22.30	30.3	37.46	38.7	21.52	21.06	31.97	23.28
Quintile 2 (vs Quintile 5)	Beta	0.11***	0.35***	0.12***	0.15***	0.23***	0.06***	0.21***	0.22***
	Contribution	-0.04***	-0.077***	-0.03***	-0.04***	-0.06***	-0.02***	-0.05***	-0.05***
	Contribution %	24.90	12.20	16.87	21.51	12.97	18.46	8.08	16.36
Quintile 3 (vs Quintile 5)	Beta	0.04	0.31***	0.07**	0.11***	0.19***	0.04***	0.12***	0.24***
	Contribution	-0.003***	-0.02***	0.000	-0.004***	-0.02***	-0.003***	-0.003***	-0.02***
	Contribution %	1.85	2.77	-0.26	1.82	3.43	3.6	0.53	5.72
Quintile 4 (vs Quintile 5)	Beta	-0.05**	0.21***	0.04	0.04*	0.10***	0.02*	0.04***	0.07**
	Contribution	-0.01***	0.04***	0.01***	0.01***	0.02***	0.006***	0.01***	0.01***
	Contribution %	8.92	-5.81	-6.17	-4.18	-4.77	-6.55	-1.7	-4.78
Married/cohabit (versus single)	Beta	0.03	0.14***	0.03*	0.06***	0.03	0.02	0.10***	0.12***
	Contribution	-0.001	-0.05***	-0.00379**	-0.01***	-0.002	0.000	-0.02***	-0.03***
	Contribution %	0.46	8.47	2.1	5.06	0.41	-0.46	3.87	9.16
Divorced/Widowed	Beta	0.04	0.07***	0.04	0.05**	0.05	-0.01	0.06***	0.12***

(versus single)									
	Contribution	-0.00	0.001***	-0.000*	0.000	-5.89**	3.90***	-0.000***	-0.002*
	Contribution %	0.08	-0.23	0.22	-0.09	0.01	-0.04	0.07	0.72
Primary/secondary (versus no-schooling)	Beta	-0.07	-0.18***	-0.09***	-0.12***	-0.15***	-0.08***	-0.17***	-0.15***
	Contribution	0.04***	-0.09***	0.03***	0.09***	-0.01***	0.05***	0.03***	0.04***
	Contribution %	-29.02	13.54	-16.24	-44.03	3.27	-51.73	-4.97	-12.12
Tertiary education (versus no-schooling)	Beta	-0.12**	-0.38***	-0.18***	-0.15***	-0.36***	-0.11***	-0.28***	-0.37***
	Contribution	-0.01***	-0.02***	-0.08***	-0.02***	-0.10***	-0.01***	-0.10***	-0.02***
	Contribution %	9.65	2.97	46.63	11.68	22.96	13.49	17.54	6.95
Currently using family planning	Beta	0.001	-0.06***	-0.02**	0.01	-0.03**	0.01	-0.01	-0.00986
	Contribution	-3.29	-5.31	0.000	0.001***	-0.003***	-0.000	-0.001***	-0.001***
	Contribution %	0.02	0.01	-0.13	-0.54	0.78	0.24	0.25	0.47
Age 20-24 (vs Age 15-19)	Beta	-0.06**	-0.06***	-0.01	0.05**	0.03	0.04**	-0.04***	-0.02
	Contribution	0.000	-0.002***	0.001***	0.001**	0.001*	-0.000	0.001*	-0.002***
	Contribution %	-0.12	0.33	-0.3	-0.6	-0.11	0.24	-0.09	0.61
Age 25-34 (versus Age 15-19)	Beta	-0.003	-0.01	0.03	0.07***	0.13***	0.061***	-0.03**	0.09***
	Contribution	-0.00	-0.001***	0.004***	0.005***	0.005*	0.000	-0.001**	0.002
	Contribution %	0.08	0.13	-2.07	-2.41	-1.16	-0.18	0.25	-0.56
Age 35-45 versus Age 15-19)	Beta	0.0452*	0.046**	0.09***	0.15***	0.21***	0.11***	0.004	0.22***
	Contribution	-0.000102	-0.005***	0.00	-0.001	0.000	0.011***	0.0001*	-0.01***
	Contribution %	0.07	0.79	-1.34	0.42	-0.02	-11.85	-0.02	4.48
Access to a flush toilet of sorts	Beta	-0.00951	0.02	0.03*	-0.005	-0.02	-0.003	-0.03**	-0.08
	Contribution	-0.0114***	0.000***	0.02***	-0.002***	-0.02***	-0.004***	-0.05***	-0.003***
	Contribution %	7.57	-0.08	-12.58	0.97	5.47	3.86	7.84	0.87
Self-report having access to place to wash hands	Beta	0.00283	-0.08***	0.05***	-0.03**	-0.01	-0.007		-0.02
	Contribution	0.00132***	-0.004***	0.01***	-0.01***	-0.02***	-0.005***		-0.005***
	Contribution %	-0.88	0.7	-4.89	7.08	3.41	4.94		1.58
Urban vs. (rural)	Beta		-0.035**	-0.06***	-0.03**	-0.08***	-0.06***	-0.06***	-0.09***
	Contribution		-0.03***	-0.09***	-0.02***	-0.06***	-0.06***	-0.10***	-0.04***
	Contribution %		5.04	49.22	11.4	14.11	67.82	0.00	12.00

*** p<0.01, ** p<0.05, * p<0.1

Table S3. Decomposition of the MHM conditions CCI .

		Kinshasa	Ethiopia	Ghana	Kenya	Rajahstan	Indonesia	Nigeria	Uganda
Quintile 1 (vs Quintile 5)	Beta	0.04**	0.07***	0.08**	0.20***	0.31***	0.21***	0.32***	0.40***
	Contribution	-0.01***	-0.03***	-0.04***	-0.08***	-0.11***	-0.061***	-0.16***	-0.16***
	Contribution %	27.67	18.80	31.51	33.12	20.39	19.48	42.33	39.82
Quintile 2 (vs Quintile 5)	Beta	0.03**	0.08***	0.12***	0.13***	0.29***	0.11***	0.22***	0.32***
	Contribution	-0.01***	-0.02***	-0.03***	-0.04***	-0.07***	-0.03***	-0.05***	-0.07***
	Contribution %	24.35	11.52	25.70	16.20	13.98	9.56	12.65	17.81
Quintile 3 (vs Quintile 5)	Beta	0.04***	0.06***	0.11***	0.05**	0.22***	0.04***	0.17***	0.32***
	Contribution	-0.003***	-0.004***	0.001	-0.002***	-0.02***	-0.004***	-0.005***	-0.02***
	Contribution %	8.79	2.27	-0.60	0.74	3.52	1.26	1.22	5.67
Quintile 4 (vs Quintile 5)	Beta	0.04***	0.03***	0.13***	0.04*	0.09***	0.02*	0.12***	0.18***
	Contribution	0.01***	0.01***	0.04***	0.01***	0.02***	0.01***	0.03***	0.04***
	Contribution %	-27.07	-3.27	-28.66	-3.22	-3.85	-2.28	-6.99	-9.003
Married/cohabit (versus single)	Beta	0.002	0.01	0.06**	-0.02	0.01	0.06***	-0.05***	-0.04*
	Contribution	-5.60	-0.005***	-0.01**	0.003***	-0.000	0.001	0.01***	0.01***
	Contribution %	0.15	2.96	5.33	-1.22	0.09	-0.47	-2.65	-2.63
Divorced/Widowed (versus single)	Beta	0.02	0.002	0.07**	-0.06**	0.02	0.08***	-0.02	-0.04
	Contribution	-6.93	4.38***	-0.001*	-0.000	-2.98**	-0.000***	0.000***	0.001*
	Contribution %	0.19	-0.03	0.58	0.10	0.01	0.01	-0.03	-0.18
Primary/secondary (versus no-schooling)	Beta	0.06**	-0.03***	-0.02	-0.04	-0.01	-0.19***	0.003	0.03
	Contribution	-0.03***	-0.01***	0.01***	0.03***	-0.001***	0.11***	-0.001***	-0.001***
	Contribution %	94.05	8.61	-6.61	-13.42	0.21	-34.70	0.15	2.06
Tertiary education (versus no-schooling)	Beta	0.05*	-0.04***	-0.05*	-0.10**	-0.07***	-0.26***	-0.06***	-0.05
	Contribution	0.006***	-0.002***	-0.025***	-0.01***	-0.02***	-0.03***	-0.02***	-0.003***
	Contribution %	-16.51	1.17	19.61	5.74	3.82	9.31	5.88	0.66
Currently using family planning	Beta	0.027***	-0.01	0.01	-0.01	-0.01	-0.01	0.00	0.01
	Contribution	-0.001	-1.27	-7.36	-0.001***	-0.002***	0.000	0.00***	0.002***
	Contribution %	1.81	0.01	0.06	0.56	0.35	-0.06	-0.09	-0.46
Age 20-24 (vs Age 15-19)	Beta	-0.03**	-0.001	-0.01	-0.001	-0.03	-0.04**	0.03*	0.003
	Contribution	7.76	-3.23***	0.000***	-3.43**	-0.000*	0.000	-0.000*	0.000***
	Contribution %	-0.21	0.02	-0.26	0.01	0.09	-0.08	0.09	-0.064
Age 25-34 (versus Age 15-19)	Beta	-0.03**	-0.001	-0.11***	0.02	-0.03	-0.03	0.04***	0.03
	Contribution	-0.001	-8.52***	-0.01***	0.001***	-0.001*	-7.02	0.002***	0.000
	Contribution %	2.75	0.05	9.17	-0.63	0.21	0.02	-0.51	-0.12

Age 35-45 versus Age 15-19)	Beta	-0.03**	0.003	-0.08***	-0.01	-0.03	-0.03	0.04**	0.07**
	Contribution	6.64	-0.000***	-0.002	4.94	-1.41	-0.003***	0.001*	-0.004***
	Contribution %	-0.18	0.22	1.78	-0.02	0.003	0.97	-0.33	1.07
Access to a flush toilet of sorts	Beta	-0.00746	-0.08***	-0.04*	-0.11***	-0.20***	-0.27***	-0.11***	-0.13***
	Contribution	-0.01***	-0.002***	-0.03***	-0.04***	-0.24***	-0.31***	-0.20***	-0.005***
	Contribution %	24.12	1.09	24.90	16.94	47.38	100.44	50.87	1.13
Self-report having access to place to wash hands	Beta	-0.01	-0.06***	-0.06***	-0.07***	-0.13***	-0.05***		0.01
	Contribution	-0.004***	-0.003***	-0.01***	-0.04***	-0.18***	-0.03***		0.003***
	Contribution %	11.40	2.14	9.50	15.10	35.34	10.13		-0.74
Urban vs. (rural)	Beta		-0.04***	0.02	0.02	0.02	-0.02	-0.04***	-0.01
	Contribution		-0.03***	0.03***	0.01***	0.01***	-0.02***	-0.06***	-0.005***
	Contribution %		21.04	-24.55	-6.22	-2.32	5.35	15.60	1.21

*** p<0.01, ** p<0.05, * p<0.1
